# How mindful of their own health are healthcare professionals? perception and practice of personnel in a tertiary hospital in Nigeria

**DOI:** 10.4314/gmj.v54i4.3

**Published:** 2020-12

**Authors:** Irikefe P Obiebi, Nnamdi S Moeteke, Godson U Eze, Ibiyemi J Umuago

**Affiliations:** 1 Department of Community Medicine, Delta State University Teaching Hospital, Oghara, Nigeria

**Keywords:** Healthcare Professionals, Self-health, Perception, Self-care, Practice

## Abstract

**Objectives:**

To assess health professionals' perception and determinants of their health and practice of preventive self-care

**Methods:**

An analytic cross-sectional design was employed, and 232 professionals were selected by stratified sampling from all health professional departments of Delta State University Teaching Hospital. Healthcare professionals who had worked in the hospital for at least six months were included in the sampling frame. Pregnant women and supernumerary professionals were excluded. A self-administered questionnaire was used, and data analysed using SPSS. The main outcome measures were the level of perception of self-health and level of practice of preventive selfcare.

**Results:**

More than four-fifths of doctors and 64.8% of nurses had good perception of their health, with significant association between perception and service area (X^2^ = 11.828, p =0.008). Screening practice was lowest amongst doctors except for HIV/HBV screening. Whereas 63.4% of all participants adjudged their BMI to be normal, only 36.2% actually had normal BMI, the difference being significant (*p* <0.001). Almost 20% of doctors had not had a BP check in a year or more, and the same proportion of doctors and nurses had never checked their FBS. The proportion of personnel who had never checked their serum lipid profile was high among nurses (76.1%) and doctors (58.3%).

**Conclusion:**

Respondents had good perception but poor preventive behaviour, beginning management after disease onset. This may be ominous for the sector. Urgent health promotion action to safeguard productivity is needed. Comprehensive data from a multi-centre study will provide a deeper understanding of the issue.

**Funding:**

None declared

## Introduction

Health is an essential social need of every human being. It has been defined by the World Health Organisation (WHO) as a state of “complete physical, social and mental well-being, and not merely the absence of disease or infirmity”.^1^ Healthcare professionals (HCPs) have been defined by the WHO as those who “study, diagnose, treat and prevent human illness, injury and other physical and mental impairments in accordance with the needs of the populations they serve. They advise on or apply preventive and curative measures and promote health with the goal of meeting the health needs and expectations of individuals and populations and improving population health outcomes. They also conduct research and improve or develop concepts, theories, and operational methods to advance evidence-based health care. Their duties may include the supervision of other health workers.^2^

According to the 2008 International Standard Classification of Occupations (ISCO) of the International Labour Organisation (ILO), Healthcare professionals (ISCO-08) consist of medical doctors, dentists, nursing professionals, midwifery professionals, pharmacists, paramedical practitioners, physiotherapists, dieticians and nutritionists, as well as traditional/complementary medicine practitioners.^2^ They provide healthcare services, guided by ethical standards, practices and techniques, to the rest of society. They are, therefore, at the forefront of ensuring that the population's health needs are sufficiently and timely met. However, HCPs are also susceptible to illnesses and diseases regardless of the fact that they have lower morbidity and mortality indices than the rest of the population.^3^ Their conditions may be complicated by a false sense of security that makes them underestimate their risk and continue with their usual activities despite deteriorating health.

This could lead them to present late for treatment when disease is already advanced and irreversible. It is uncertain how this important group of workers look after their own health.^4^ WHO has defined “self-care” as “the ability of individuals, families and communities to promote health, prevent disease, maintain health, and to cope with illness and disability with or without the support of a health-care provider”. The scope of self-care includes health promotion, disease prevention and control, seeking hospital/specialist care if necessary, and rehabilitation including palliative care.^5^

Some authors have reported that when health care professionals take ill, some tend to neglect valid expert counsel given to lay patients and have assumed an attitude of denial as a defence mechanism. They occasionally resort to self-diagnosis, self-prescription and self-medication when the situation gets unbearable.^6^,^7^ In spite of warnings from standard medical practice against self-medication, it is alarming to discover that some health care workers including physicians think it is appropriate to self-medicate and have investigations done aside proper protocol.^8^ For those who eventually seek aid from other health professionals, they often get inadequate and poor care because they employ the services of non-specialists and those who do not have adequate skills and experience.^8^ On the contrary, other studies suggest that healthcare practitioners seek care more than the general population^8^ and more readily have regular physical examination (blood pressure, breast examination, etc.) and investigations (such as cholesterol level, prostatic specific antigen and fasting blood sugar) done at the appropriate time.^9^,^10^ Many health workers work long hours each day and tend to have little or no time for adequate sleep and regular exercise.^10^ They are faced with stressful conditions daily, both at work and home, and are not exempt from illhealth, 11 particularly because work-related stress is directly linked to poor health.^12^Also, many do not consider seeing a physician except when they fall ill.^13^ Thus, they do not access and utilise health-promoting and preventive services. A sizeable number of health workers suffer from long-term illnesses affecting various systems of the body- cardiovascular, respiratory, musculoskeletal, etc.^14^–^16^ A large number of healthcare workers die as a result of diseases ranging from cardiovascular, respiratory, and oncologic to psychiatric illnesses but only a few healthcare workers promptly obtain health care.^17^–^19^

The wellbeing of health care professionals should be of utmost importance so that they can continuously render the needed services to the increasing number of people who visit health facilities. Incidentally, most of the recent literature on the health of healthcare workers is centred on psychiatric or mental disorders (for which they, especially physicians, have been shown to have a higher risk than the average person) 20,21 whereas most deaths are due to physical illnesses just as in the general population.^22^ Physicians “do not practice personal preventive medicine; and do not seek, or delay seeking, appropriate help when they experience illness or distress”.^20^ These studies have mainly been done in the western world, and data on how this group of workers in Nigeria look after their health is very limited.^12^,^23^ Furthermore, there has been an upsurge in reports of sudden unexplained natural deaths among HCPs in Nigeria. This study was conducted to assess how HCPs perceive their health, and to ascertain their health screening practices and determinants with the hope that it would provide relevant data to inform policy, stimulate behavioural change and close any identified gaps in practice.

## Methods

### Study Area and Design

A cross-sectional study was conducted among HCPs in Delta State University Teaching Hospital (DELSUTH), Oghara, Delta State, Nigeria.

### Sample Size/Sampling Technique

The Cochrane formula for calculating minimum sample size for prevalence studies was used: $n=\frac{z^{2}(pq)}{d^{2}}$ where z = 1.96 (i.e. standard normal deviate at 95% confidence limit); p= 26% being the proportion of doctors with health problems but felt there was no need to see a physician^18^; q = 1-p (1 – 0.26 = 0.74); d = error margin of 5% (0.05); n = 296.

Since the population of HCPs in DELSUTH is less than 10,000 we used a correction factor:

$$N=\frac{n\;Pt}{n+(Pt-1)}=200$$

where n = 296; and Pt = total population of HCPs in DEL-SUTH (623).

To compensate for an anticipated non-100% response rate, the formula below was used: Sample size (N) = calculated sample size (200) ÷ anticipated 90% response rate (0.9). Therefore, estimated minimum sample size was 223.

All HCPs who had been employed fulltime in the hospital for at least six months were included in the sampling frame. Pregnant women and supernumerary professionals were excluded. Two hundred and thirty-two (232) HCPs comprising doctors (96); nurses (88); and physiotherapists, laboratory scientists and pharmacists (48) were selected by stratified sampling technique from the different health professional departments in the hospital: medical/surgical (Internal Medicine, Paediatrics, Surgery, Obstetrics & Gynaecology, Accident & Emergency, etc), nursing, diagnostic (Radiology and Laboratory), and support (physiotherapy and pharmacy), based on the number of HCPs in the individual departments, the total in the hospital and the calculated minimum sample size.

### Data Collection/Analysis

Data collection was done between October and December 2016. A pre-tested, semi-structured self-administered questionnaire was used, with a section for entry of values of anthropometric measurements which were done and filled-in by the investigators. Questions were asked to ascertain knowledge of timing of health checks; family history of diseases; personal blood group, haemoglobin genotype, and HIV status; etc. Right and Wrong responses attracted a score of 2 and 1 respectively; no response had a score of 0. Total score was 14. The 25^th^ percentile score was 12. Poor perception of self-health was set at score <12; and good perception, ≥12. Respondents indicated what they perceived as their Body Mass Index (BMI) category. This was matched against their actual BMI. Wrong matches were categorised as ‘wrong perception of BMI’ while correct matches were categorised as ‘right perception’. A stadiometer with a measuring range of 50–205cm and a graduation of 1mm (1/8′) was used to ascertain the heights of participants, while an electronic weighing scale (Harrison Emperors made in the People's Republic of China) with a maximum capacity of 120kg in divisions of 100 grams was used to measure body weight as prescribed by the National Health and Nutrition Examination Survey (NHANES) III protocol.^24^ BMI for each participant was obtained by dividing their weight (Kg) by the square of height (metres).

The data collected were analysed with the Statistical Package for Social Sciences (SPSS) version 22 software. Categorical data such as education, marital status, professions, and age groups were expressed in percentages while continuous variables such as ‘time spent on physical exercise or in front of a television’ were summarised using mean and standard deviation. Hypotheses were tested with chi-square and student *t* tests. Magnitude of association between determinants (explanatory variables such as sex, age group, profession, years of work, marital status, etc) and outcomes (such as perception of selfhealth, perception of actual BMI, and screening practice) was determined using odds ratio. The level of significance was set at alpha (*α*) less than 0.05.

### Ethical Consideration

Approval to conduct this study was given by the Health Research Ethics Committee (HREC) of Delta State University Teaching Hospital with ID DELSUTH/HREC/2015/025.

Written informed consent was obtained from each participant before recruiting them for this study. Study participants were not asked for their names or any information that could reveal personal identity.

## Results

The mean age of respondents was 35.19 ± 5.68 years while the modal (62.9%) age group was 31–40 years. Doctors and nurses comprised 41.4% and 37.9% of respondents, respectively. Three-quarters of respondents (174, 75%) were married, 48 (20.7%) were single and not cohabiting, 6 (2.6%) were single and cohabiting, while 4 (0.4%) were separated/divorced/ widowed. Christians made up 98.7% of the sample.

Almost three-quarters of respondents (74.1%) had a good perception of health; there was no difference between males and females in this regard. However, the highest proportion of HCPs with good perception (83.3%) was found among doctors. About two-thirds of nurses (64.8%) had good perception of health compared to only half of ‘other HCPs’, *p=0.016*. The majority (80%) of respondents with chronic medical conditions had a good perception as against a slightly lower proportion among persons without chronic medical conditions. Almost everyone with a chronic medical condition (92.0%) was having it managed (Table 1).

**Table 1:** Potential predictors of overall perception of self-health

		Perception of self-health Frequency (%)		
Variable	Category	Poor n = 60	Good n = 172	Total N = 232
**Sex**	Male	31 (26.5))	86 (73.5)	117 (100.0)
	Female	29 (25.2)	86 (74.8)	115 (100.0)
	***X*^2^ = 0.049, p=0.824; OR: 1.070 (0.590 – 1.920)**			
**Age Group**	21–30	17 (31.5)	37 (68.5)	54 (100.0)
	31–40	35 (24.0)	111 (76.0)	146 (100.0)
	>41	8 (25.0)	24 (75.0)	32 (100.0)
	***X*^2^ = 1.174, p=0.556**			
**Profession**	Doctors	16 (16.7)	80 (83.3)	96 (100.0)
	Nurses	31 (35.2)	57 (64.8)	88 (100.0)
	*Others	13 (50.0)	35 (50.0)	48 (100.0)
	***X^2^ = 8.296, p = 0.016***			
**Duration of Professional** **work**	1–10	46 (24.0)	146 (76.0)	192 (100.0)
	11–20	12 (33.5)	22 (66.5)	34 (100.0)
	>20	2 (33.3)	4 (66.7)	6 (100.0)
	***X^2^=2.115, p=0.347***			
**Service area**	Medical/surgical	15 (15.0)	85 (85.0)	100 (100.0)
	Nursing	31 (35.2)	57 (64.8)	88 (100.0)
	Diagnostic	9 (28.1)	23 (71.9)	32 (100.0)
	Support	5 (41.7)	7 (58.3)	12 (100.0)
	***X^2^ = 11.828, p =0.008***			
**Currently married**	Yes	48 (31.5)	126 (68.5)	174 (100.0)
	No	12 (24.0)	46 (76.0)	58 (100.0)
		***X^2^* = 1.585, *p* = 0.300; *OR:* 1.460 (0.710 – 2.990)**		

Others: Pharmacists, Lab scientists, physiotherapists

* Likelihood ratio Chi-square

Whereas over three-fifths (63.4%) of all participants adjudged their BMI to be normal, only about a third (36.2%) had a normal BMI based on objective measurements. The difference in proportion between perceived and actual BMI was highly significant (p<0.001). Respondents with a normal BMI had the least tendency to be wrong about their BMI category while the obese had the most tendency to be wrong about their category (p<0.001). Females were significantly more likely to be wrong about their BMI, p=0.003; the odds of their having a wrong perception of BMI was more than twice that of males, OR=2.24 (CI: 1.32 – 3.80). Wrong assumption of BMI was significantly higher among married persons, and increased with years of work, p = 0.048, and 0.019, respectively. Doctors were more likely to have a right perception of their BMIs while nurses were the least likely, p<0.001(Table 2).

**Table 2:** Predictors of perception of BMI

			Frequency (%)	
Variable	Category	Wrong n = 106	Right n = 126	Total N = 232
**Sex**	Female	64 (55.7)	51 (44.3)	115 (100.0)
	Male	42 (34.9)	75 (64.1)	117 (100.0)
	***X*^2^ = 9.120, *p* = 0.003; OR = 2.24 (CI:1.32 -3.80)**			
**Age Group**	21–30	28 (59.1)	26 (40.9)	54 (100.0)
	31–40	59 (40.4)	87 (59.6)	146 (100.0)
	≥41	19 (59.4)	13 (40.6)	32 (100.0)
	***X^2^ = 4.881, p = 0.087***			
**Profession**	Doctors	34 (35.4)	62 (64.6)	96 (100.0)
	Nurses	50 (56.8)	38 (43.2)	88 (100.0)
	Others	22 (45.8)	26 (54.2)	48 (100.0)
	***X^2^ = 1.585, p = < 0.001***			
**Duration of Professional** **work**	1–10	82 (42.7)	110 (57.3)	192 (100.0)
	11–20	20 (58.8)	14 (41.2)	34 (100.0)
	>20	4 (66.7)	2 (33.3)	6 (100.0)
	***X^2^= 7.933, p=0.019***			
**Service area**	*Med/Surg.	35 (35.0)	65 (65.0)	100 (100.0)
	Nursing	50 (56.8)	38 (43.2)	88 (100.0)
	Diagnostic	14 (43.7)	18 (56.3)	32 (100.0)
	Support	7 (41.7)	5 (58.3)	12 (100.0)
	***X^2^ =9.819, p = 0.020***			
**Currently married**	Yes	86 (49.4)	88 (50.6)	174 (100.0)
	No	20 (24.0)	38 (76.0)	58 (100.0)
	***X^2^* =3.910, *p* = 0.048; *OR*: 1.46 (1.00–3.44)**			
**Sitting for long** **hours at work in a** **typical day**	Yes	39 (44.3)	28 (55.7)	67 (100.0)
	No	87 (64.1)	78 (34.9)	165 (100.0)
	***X^2^ =0.557, p*= 0.447; OR = 1.25 (CI:0.70 – 2.22)**			

* Med/Surg: Medical/Surgical

Number of hours spent sitting at work or days of walking/exercising per week did not vary significantly between males and females; however, males spent an average of 1.5 hours more watching TV daily compared to females. The difference was statistically significant; p<0.001 (Table 3).

**Table 3:** Sex of respondents and physical activity

Variable	Male (n=117) mean ± SD	Female(n=115) mean ± SD	Mean diff	95% *CI		*p*- value
				Lower limit	Upper limit	
**Days walking/week**	2.52 ± 0.70	2.32 ± 0.79	0.20	-0.031	0.424	0.09
**Hours sitting at work**	5.06 ±4.47	4.4 ± 4.54	0.659	-0.476	1.794	0.250
**Days exercising/week**	1.9 ± 0.87	1.83 ± 0.94	0.072	-0.242	0.385	0.652

Four-fifths of respondents (80.6%) had checked their weights within the six months prior to the study. Practice of weight checks did not differ between different groups of HCPs. However, their practice of Blood Pressure (BP) checks differed significantly; it was best amongst nurses and worst among doctors, p=0.007. Only 4.6% of nurses had never had a blood pressure check or not done so in the last one year, in contrast to almost a fifth (19.8%) of doctors who had not had a BP check in a year or more. Although practice of having Fasting Blood Sugar (FBS) check did not differ significantly between all HCPs, nearly a fifth of doctors and nurses had never had FBS checks, while about half (51.0%) of doctors and about two-thirds (65.8%) of nurses did their last FBS less than a year prior to the survey. Lipid profile check was significantly lower among nurses compared to other health workers, p<0.001. Over three-quarters (76.1%) of nurses and over half (58.3%) of doctors had never done a lipid profile check.

Slightly over two-fifths (41.7%) of ‘other HCPs’ compared to a quarter (25.0%) of nurses had Hepatitis B virus (HBV) screening less than six months earlier. Almost equal percentages of each group of HCPs had never screened for HBV or last screened over a year; *p=0.181*. Almost the same proportions of nurses and doctors (35.7% versus 35.0%) had an eye check in the previous one year, whereas a significantly higher proportion of ‘other HCPs’ (39.6%) had never had an eye check, *p*=0.028. Over half of the nurses (56.8%) and about a third (34.4%) of doctors had never had a dental check, while about a quarter of doctors and other health workers, except nurses, visited a dentist in the year preceding this study. The practice of having dental check-ups differed significantly between HCPs, p = 0.032 (Table 4).

**Table 4:** Respondents' screening practice by profession

		Profession of Respondents Frequency (%)		
Variable	Category	Doctors(n=96)	Nurse(n=88)	Others (n=48)
**Last Weight Check**	<6 months	77 (80.2)	73 (82.9)	37 (77.1)
	≥6months/never	19 (19.8)	15 (17.1)	11 (22.9)
	*X^2^ = 0.701, p= 0.704			
**Last BP Check**	<1 year	77 (80.2)	84 (95.4)	40 (83.3)
	≥1 year/never	19 (19.8)	4 (4.6)	8 (16.7)
	*X^2^ = 9.790, p= 0.007			
**Last FBS Check**	<1 year ago	49 (51.0)	49 (65.8)	30 (62.5)
	≥1years	29 (30.2)	23 (26.1)	10 (20.8)
	Never	18 (18.8)	16 (18.1)	8 (16.7)
	*X^2^ =1.916, p= 0.751			
**Last Lipid Profile**	<3 years ago	30 (31.3)	15 (17.1)	25 (52.1)
	≥3years	10 (10.4)	6 (6.8)	3 (6.2)
	Never	56 (58.3)	67 (76.1)	20 (41.7)
	*X^2^ = 19.981, p= <0.001			
**Last HIV Screening**	<6 months ago	42 (43.8)	38 (43.2)	25 (52.1)
	≥6 months ago	50 (52.1)	47 (53.4)	18 (37.5)
	Never	4 (4.2)	3 (3.4)	5 (10.4)
	*X^2^ = 5.689, p= 0.223			
**Last HBV Screening**	<6 months ago	27 (28.1)	22 (25.0)	20 (41.7)
	≥6 months	57 (59.4)	52 (59.1)	19 (39.6)
	Never	12 (12.5)	14 (15.9)	9 (18.7)
	*X^2^ = 6.603, p= 0.158			
**Last Eye Check**	<1 year ago	39 (35.0)	31 (35.7)	13 (27.1)
	≥1 year ago	39 (14.5)	20 (19.1)	16 (33.3)
	Never	18 (35.0)	27 (20.0)	19 (39.6)
	*X^2^ = 10.826, p= 0.028			
**Last Dental Check**	<1 year ago	24 (25.0)	13 (14.8)	13 (27.1)
	≥1 year ago	39 (40.6)	25 (28.4)	15 (31.2)
	Never	33 (34.4)	50 (56.8)	20 (41.7)
	*X^2^ =10.550, p= 0.032			

* Yates corrected

The practice of screening was better among females for all tests except for HIV and HBV screening for which a higher proportion of males had been screened recently. These differences were however statistically significant in favour of females, only for BP and eye checks, p=0.013 and 0.018 respectively; while males screened significantly more recently for HIV, p=0.004.

Overall, a higher proportion of males had never screened for all tests profiled in this study. More than four-fifths of both male and female respondents have had both BP and weight checks in the last one year; less than a tenth of females did not check their BPs in the last year prior to the study. Serum lipid profile was the least performed test among all screening tests profiled: over three-fifths of both males and females had never checked it.

Only a little over a fifth of all respondents had never screened for HIV (Table 5).

**Table 5:** Practice of screening and sex of health workers

	Category	Sex of Respondents Frequency (%)		
		Male (n=117)	Female (n=115)	Total (N= 232)
**Last weight check**	<6 months	93 (79.5)	99 (86.1)	192 (82.8)
	≥6 months	24 (20.5)	16 (13.9)	40 (17.2)
	X^2^ = 1.338*, p= 0.247			
	<1 year	95 (81.2)	106 (92.2)	201 (86.6)
	≥1 year/ Never	22 (18.8)	9 (7.8)	31 (13.4)
	X^2^ = 6.115*, p= 0.013			
	<1 year	56 (47.9)	72 (62.6)	128 (55.2)
	≥1 year	37 (31.6)	25 (21.7)	62 (26.7)
	Never	24 (20.5)	18 (15.7)	42 (18.1)
	X^2^ = 5.163, p= 0.076			
**Last Lipid Profile Check**	<3 years	33 (28.2)	38 (33.0)	71 (30.6)
	≥3 years	10 (8.6)	8 (7.0)	18 (7.8)
	Never	74 (63.2)	69 (60.0)	143 (61.6)
	X^2^ = 0.734, p= 0.694			
**Last HIV Screen**	<6 months	63 (53.8)	42 (36.5)	105 (45.3)
	≥6 months	45 (38.5)	69 (60.0)	114 (49.1)
	Never	9 (7.7)	4 (3.5)	13 (5.6)
	X^2^ = 11.159, p= 0.004			
**Last HBV Screen**	< 1 year	72 (61.5)	67 (58.3)	139 (59.9)
	≥1 year	25 (21.4)	32 (27.8)	57 (24.6)
	Never	20 (17.1)	16 (13.9)	36 (15.5)
	X^2^ = 1.467, p= 0.480			
**Last Eye Check**	<1 year	41 (35.0)	41 (35.7)	82 (35.3)
	≥ 1 year	35 (30.0)	51 (44.3)	86 (37.1)
	Never	41 (35.0)	23 (20.0)	64 (27.6)
	X^2^ = 8.023, p= 0.018			
**Last Dental Check**	<1 year ago	23 (19.7)	27 (23.5)	50 (21.6)
	≥ 1 year	35 (29.9)	43 (37.4)	78 (33.6)
	Never	59 (50.4)	45 (39.1)	104 (44.8)
	X^2^ = 3.008, p= 0.222			

## Discussion

The good perception of health which was noted amongst most respondents was expected given that it was a largely young population, the majority being in their third and fourth decades of life – a period when chronic disease is not usually expected. In a similar study conducted in Barcelona, respondents displayed unsatisfactory attitudes towards self-healthcare. This may have been more obvious as only doctors participated in that study.^25^ In the index study, doctors also had the best perception of health but lagged behind nurses in their actual practice of screening for most of the tests profiled. Doctors usually lead the medical team, and as such, tend to see themselves as the ultimate in healthcare and would rather self-diagnose and treat as they deem fit than seek medical help from others.^26^ Doctors had a better insight about health probably because they usually have higher level of training. Also, workers in clinical service areas were more likely than those in support services to have a right perception. This finding is not isolated as it is likely that support staff, not being in mainstream patient care, may have less knowledge about healthcare and so aligned their attention towards other interests.

It is also not unlikely that providers of clinical services have in their curricula guidelines for adopting healthy behaviours such that healthy practices have become imbued as an integral part of their lifestyle.

Over a fifth of all respondents were obese but less than a fifth of these obese persons perceived themselves to be so, showing that a wrong perception of body image is common even among healthcare professionals.^27^. Health workers are expected to be more knowledgeable and should be able to tell whether their weight is normal or not.^26^ This observation mirrors a common societal norm where people underestimate their body weight, perhaps due to a ‘*feel-good’* attitude which seeks to shut out negative emotions. Experiencing weight-based discrimination is possibly linked to mental dysfunction.^28^,^29^ Since weight-associated stigma is becoming prevalent in societies, the study participants may have deliberately misreported their perception of their BMI as a coping strategy to avoid the psychological stress from supposed weight-related prejudice. A study in Israel reported that over half of the nurses in obstetrics and gynaecology perceived themselves to be overweight or obese and had to adopt special diet regimens to reduce weight.^30^ Compared with others, more nurses in our study had a wrong perception of their weight. Unlike the Israeli nurses, they perceived their BMIs as lower than they actually were. Females were twice as likely as males to have a wrong perception of their BMI, probably because it is ladies who are more concerned about their physical appearance.

Obesity is an identifiable medical disorder with a social stigma capable of undermining people's health and well-being with a resultant intensified drive to evade scrutiny. People may attempt to break free from the humiliation by adopting unwholesome weight-reducing actions.^31^ The prevalence of obesity is becoming increasingly high such that over three-fifths (61.6%) of medical staff in this study were overweight or obese. The tendency to have a right perception about body weight decreased with the duration of professional experience. This observation is not only unexpected but noteworthy. There may be no clear-cut explanation except that HCPs, like other people, are less mindful of their weight as they advance in age. Health workers in medical and surgical service areas were more likely than those in nursing or supportive services to have a right perception. As nurses and other support staff were predominantly females, this difference is likely a reflection of the overarching peculiarity of females in this study.

Males were more disposed to lead a sedentary lifestyle as they spent higher hours sitting at work and in front of the television. This finding may be a reflection of how male HCPs look after their health and is congruent with that from a similar study where being a male doctor was associated with higher health risks.^26^ The average number of days for which participants exercised or walked was less than three days per week, and is sub-optimal for cardiovascular health, and inadequate to ward off cardiovascular events. It is known that sustained physical activity has a major role in preventing death even amongst persons with ischaemic heart disease.^32^ Besides the mortality associated with physical inactivity, healthcare workers are seen as role models for physical activity.^33^ As a consequence, they need to reflect on their own practice of physical exercise and make necessary adjustments. Health workers should not only have right knowledge but should also practice what they advocate.^34^ The fact that almost all respondents with a chronic ailment were on treatment suggests respondents are quick to act only after they have been diagnosed with a condition. This is in disagreement with previous studies that concluded that doctors in Nigeria 35 and the United Kingdom 26 have an unsatisfactory attitude and are hesitant towards seeking care for themselves when ill.

As seen in similar studies^36^–^38^, uptake of HIV testing was the utmost as only 1 in 20 HCPs were yet to screen. While this appears laudable, no HCP can be excused in the drive to achieve the goals of the HIV prevention policy.^39^ Nurses surpassed Doctors in screening for two diseases (hypertension and diabetes) with high prevalence in our setting.^40^ Though doctors' level of self-reported Lipid profile, HBV and dental screening improved, these tests were still more predominant among ‘other HCPs’. Although a likely basis for this screening disparity is not readily evident, it suffices to say that, relatively, doctors probably ignore the importance of screening. Differential attitude towards screening has been documented amongst HCPs in a different tertiary hospital.^38^ Generally, males were proportionately less likely to have screened for all tests although, more males screened for HIV and HBV in the recent months preceding this study. Other studies have demonstrated poorer self-care amongst men.^41^–^44^

## Conclusion

The findings of this study indicate a shortfall in self-care among HCPs. Though they had good perception of self-health, the practice of preventive self-care was absent in significant proportion of respondents, HCPs tended to begin management chronic illnesses only after disease onset. This may be ominous for the health sector. Therefore, urgent health promotion action, on the part of government and managers of the health system, to safeguard productivity in the sector is recommended. Given the limitations of this small-scale study, we suggest further research involving a multiple centre and the different levels of health care to provide comprehensive data needed for a deeper understanding of the issue.
